# Relationship between multimorbidity, functional limitation, and quality of life among middle-aged and older adults: findings from the longitudinal analysis of the 2013–2020 Survey of Health, Ageing, and Retirement in Europe (SHARE)

**DOI:** 10.1007/s11136-023-03508-9

**Published:** 2023-09-30

**Authors:** Piotr Wilk, Maria Ruiz-Castell, Saverio Stranges, Torsten Bohn, Guy Fagherazzi, Kathryn Nicholson, Valérie Moran, Tatjana T. Makovski, Maria Noel Pi Alperin, Maurice P. Zeegers, Hanen Samouda

**Affiliations:** 1 Department of Epidemiology and Biostatistics, Western University, London, Canada; 2 Institute of Social and Preventive Medicine, University of Bern, Bern, Switzerland; 3 Department of Epidemiology, Maastricht University, Maastricht, The Netherlands; 4 Department of Precision Health, Luxembourg Institute of Health, Strassen, Luxembourg; 5 Living Conditions Department, Luxembourg Institute of Socio-Economic Research, Esch-sur-Alzette, Luxembourg

**Keywords:** Multimorbidity, Quality of life, Ageing, SHARE, Longitudinal study, Latent growth curve, Elderly

## Abstract

**Purpose:**

The increased burden of multimorbidity is restricting individuals’ ability to live autonomously, leading to a poorer quality of life. This study estimated trajectories of functional limitation and quality of life among middle-aged (ages 50 to 64 years) and older (aged 65 years and older) individuals with and without multimorbidity. We also assessed differences in the relationship between these two trajectories by multimorbidity status and separately for each age cohort.

**Methods:**

Data originated from the Survey of Health, Ageing, and Retirement in Europe (SHARE). In Luxembourg, data were obtained between 2013 and 2020, involving 1,585 respondents ≥ 50 years of age. Multimorbidity was defined as a self-reported diagnosis of two or more out of 16 chronic conditions; functional limitation was assessed by a combined (Instrumental) Activities of Daily Living (ADL/IADLI) scale; and to measure quality of life, we used the Control, Autonomy, Self-Realization, and Pleasure (CASP-12) scale. Latent growth curve modelling techniques were used to conduct the analysis where repeated measures of quality of life and functional limitation were treated as continuous and zero-inflated count variables, respectively. The model was assessed separately in each age cohort, controlling for the baseline covariates, and the estimates from the two cohorts were presented as components of a synthetic cohort covering the life course from the age of 50.

**Results:**

Middle-aged and older adults living with multimorbidity experienced poorer quality of life throughout the life course and were at a higher risk of functional limitation than those without multimorbidity. At baseline, functional limitation had a negative impact on quality of life. Furthermore, among middle-aged adults without multimorbidity and older adults with multimorbidity, an increase in the number of functional limitations led to a decline in quality of life. These results imply that the impact of multimorbidity on functional limitation and quality of life may vary across the life course.

**Conclusion:**

Using novel methodological techniques, this study contributes to a better understanding of the longitudinal relationship between functional limitation and quality of life among individuals with and without multimorbidity and how this relationship changes across the life course. Our findings suggest that lowering the risk of having multimorbidity can decrease functional limitation and increase quality of life.

**Supplementary Information:**

The online version contains supplementary material available at 10.1007/s11136-023-03508-9.

## Background

Multimorbidity, commonly defined as the co-existence of two or more chronic conditions [1, 2], is affecting an increasing number of individuals worldwide [1, 3, 4]. Data from the Survey of Health, Ageing, and Retirement in Europe (SHARE) indicate that the prevalence of multimorbidity in Europe in middle-aged and older adults increased from 38% in 2006 to 42% in 2015 [5]. This trend is due to increasing life expectancy and population ageing, changes in lifestyle, and improvements in the detection of chronic conditions [6, 7]. Systematic reviews have suggested that the burden of multiple chronic conditions is associated with poorer quality of life [5, 8–11], one of the core outcome measures for multimorbidity research and healthy ageing [12]. However, little is known about the nature of the relationship between multimorbidity and quality of life [13–15]. A promising area of research has focused on the role of functional limitation [13, 15, 16], defined as the restriction of the ability of an individual to live autonomously, without physical or psychological limitations in daily activities [17, 18]. Individuals living with multimorbidity have been found to have, in addition to poorer quality of life, a higher number of functional limitations [11, 19–23]. However, due to a shortage of longitudinal prospective studies, there is limited knowledge on how multimorbidity affects functional limitation and quality of life across the life course. Specifically, we do not know if the cross-sectional associations between multimorbidity, functional limitation, and quality of life reported in the literature are persistent over time or how the increase in the degree of functional limitation among individuals living with multimorbidity affects their quality of life. A more in-depth understanding of these complex and dynamic relationships would help us to identify the mechanism by which multimorbidity impacts quality of life or how it moderates the relationship between functional limitation and quality of life across the life course. Such knowledge could potentially lead to the identification of routes and windows of opportunity by which interventions targeting individuals affected by multimorbidity could better maintain or improve their quality of life, for instance, by addressing some of the negative consequences associated with the burden of functional limitation.

The objective of the present study was to address some of these gaps by assessing how multimorbidity affects trajectories of functional limitation and quality of life and how the prospective relationship between these two trajectories is moderated by multimorbidity status and how it differs across the life course. We hypothesized that, across the life course, adults with multimorbidity would have a higher degree of functional limitation and poorer quality of life. We also predicted that an increase over time in functional limitation would produce a decline in quality of life, especially among individuals with multimorbidity. Finally, we hypothesized that the nature of the relationship between multimorbidity, functional limitation, and quality of life would differ across the life course.

## Participants and methods

### Data and study participants

SHARE is a longitudinal population-based survey of Europeans aged 50 years and older that was initiated in 2004 (wave 1) [24]. SHARE sampling and recruitment strategies were designed to derive nationally representative samples, and standardized questionnaires were translated by each SHARE participating country from an English version into national languages (i.e., French and German in Luxembourg) [25]. Face-to-face interviews were conducted using computer-assisted personal interviewing (CAPI) techniques. SHARE data contain information on respondents’ demographic, socio-economic, and health characteristics. More information about the survey design, data structure, and response rates has been previously published [24].

The data used in our study came from 1585 SHARE respondents from Luxembourg who were 50 years of age or older in 2013 at wave 5 (the baseline SHARE survey) [26]. These individuals were followed over a period of approximately seven years in three follow-ups: in 2015 (wave 6) [27], in 2017 (wave 7) [28], and in 2019–2020 (wave 8) [29]. To test if the prospective association of multimorbidity with functional limitation and quality of life differs across the life course, following Erikson’s Psychosocial Stages of Development theory [30], we divided this sample into two age cohorts: a “middle age” cohort (ages 50 to 64 years at baseline) and an “older age” cohort (aged 65 years and older at baseline). These two cohorts can be treated as components of a synthetic cohort covering the life course from the age of 50.

### Measurements

At baseline, SHARE respondents were asked if they were diagnosed with, treated for, or bothered by any of the following chronic conditions: *heart attack*, *hypertension*, *high cholesterol*, *stroke or cerebral vascular disease*, *diabetes or high blood sugar*, *chronic lung disease*, *cancer*, *stomach or duodenal ulcer, Parkinson disease, cataracts, hip or other fractures, Alzheimer’s disease or dementia*, *affective or emotional disorders*, *rheumatoid arthritis*, *osteoarthritis,* or any other conditions. Respondents with two or more of these conditions were categorized as having multimorbidity [1, 2, 31].

At each wave, respondents were asked if, because of a physical, mental, emotional, or memory problem, they have had any difficulty, lasting more than three months, with the following six Activities of Daily Living (ADL): (1) dressing, (2) walking across a room, (3) bathing or showering, (4) eating, (5) getting in or out of bed, and (6) using the toilet; or with the following seven Instrumental Activities of Daily Living (IADL): (1) using a map to figure out how to get around, (2) preparing a hot meal, (3) shopping for groceries, (4) making telephone calls, (5) taking medications, (6) doing work around the house, and (7) managing money. The summated ADL/IADL score ranges from 0 to 13, with a higher score indicating a higher degree of functional limitation [32, 33].

To measure quality of life at each wave, we used the Control, Autonomy, Self-Realization, and Pleasure (CASP-12) scale, which consists of 12 items scored on a 4-point Likert scale [34]; the total score ranges from a minimum of 12 to a maximum of 48 with a higher score indicating better quality of life. We selected this validated scale since it is an appropriate tool to measure quality of life in older adults [13], it was designed to measure positive experiences in older age, instead of focusing on negative health outcomes [35], and it was used in past research on the effects of multimorbidity and functional limitation on quality of life [13]. The Cronbach’s alphas for the four waves were 0.76, 0.78, 0.79, 0.79 for the French language questionnaire and 0.75, 0.74, 0.73, 0.72 for the German language questionnaire, respectively, indicating reasonably strong levels of internal consistency.

We also used the following baseline control variables: age differences among respondents within each age cohort (in years); gender (men vs. women); living with a partner (yes vs. no); educational attainment (secondary or less vs. post-secondary or more); household income (adjusted for household size and log transformed); and whether or not respondents were born in Luxembourg (vs. immigrants), as Luxembourg has the largest proportion of immigrants in the European Union [36].

### Data management

**Fig. 1:**
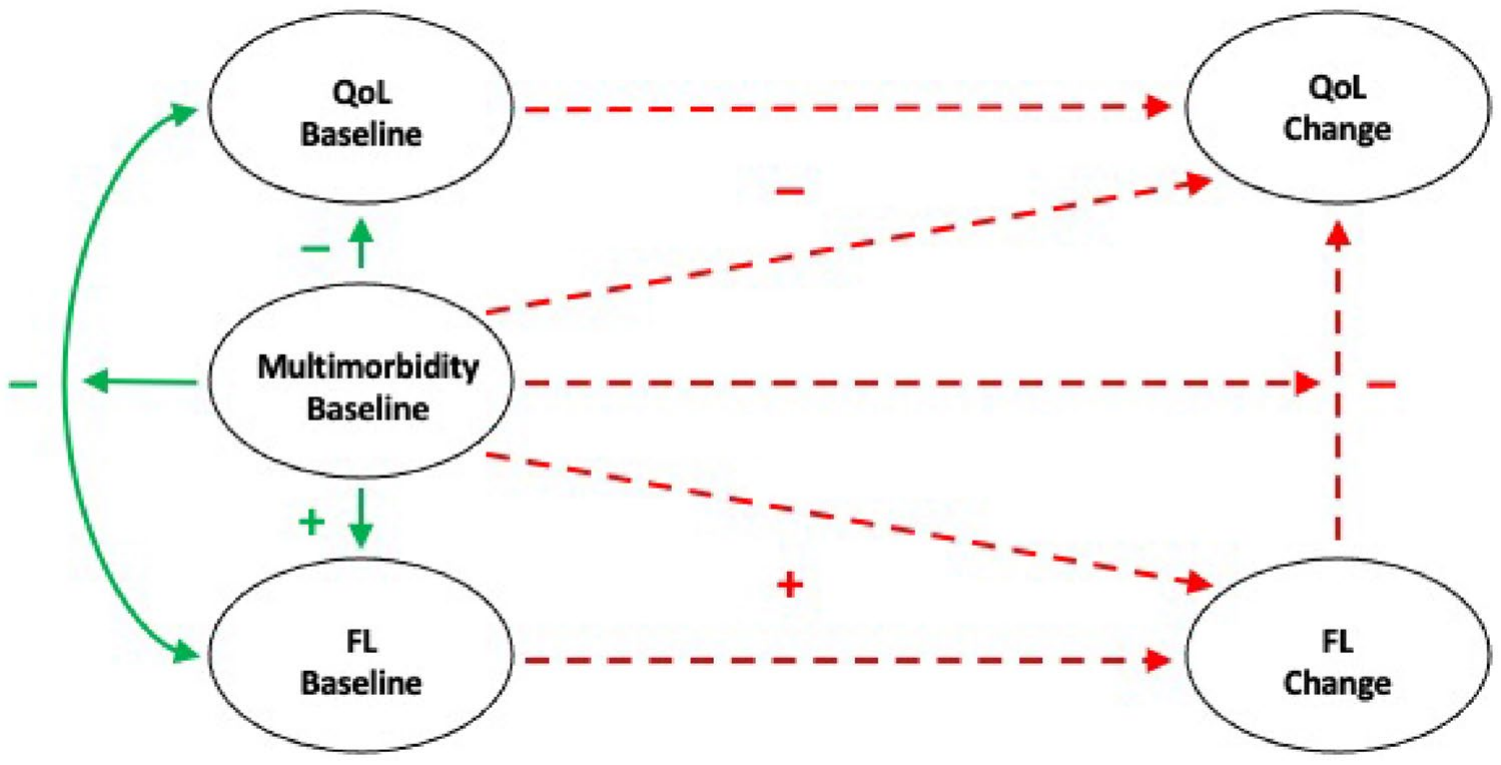
Outline of the conceptual model**.** QoL: Quality of Life. FL: Functional Limitation. Solid (green) line (—): Relationship established in the past literature. Dotted (red) line (–-): Relationships assessed in the current project

Out of 1,585 baseline respondents, 543 (34.3%) participated in all three follow-ups, 391 (24.7%) dropped out after the baseline survey, 274 (13.7%) after the first follow-up, 270 (17.0%) after the second follow-up, and 107 (6.8%) skipped the first or second follow-up but participated in the third follow-up. Overall, out of 6,348 potential time-person data points, 4,187 (66.0%) were available for the analysis while 2,161 (30.7%) were lost due to survey non-response, including 212 (3.3%) due to mortality. Supplementary Table S1 indicates that, at baseline, respondents who did not participate in all follow-ups, including those who died, were more likely to have functional limitation and had a higher number of limitations, were less likely to live with a partner or to have a higher level of education, compared to respondents who participated in all three follow-ups. In addition, repeated measures for quality of life and functional limitation had some missing data due to item non-response (between 0.1% and 5.4%). Missing data for household income were imputed by the SHARE project and may be affected by selection bias. Our analyses included all 1,585 baseline SHARE respondents, 897 (56.6%) from the “middle age” cohort and 688 (43.4%) from the “older age” cohort, regardless of their dropout pattern and item non-response. We used the full information maximum likelihood (FIML) techniques to model missing data as a function of baseline covariates and all available repeated measures based on the explicit assumption that the missing data are missing at random [37]; if the missing data do not meet this assumption, the results may be biased (Fig. 1).

### Statistical analysis

**Fig. 2:**
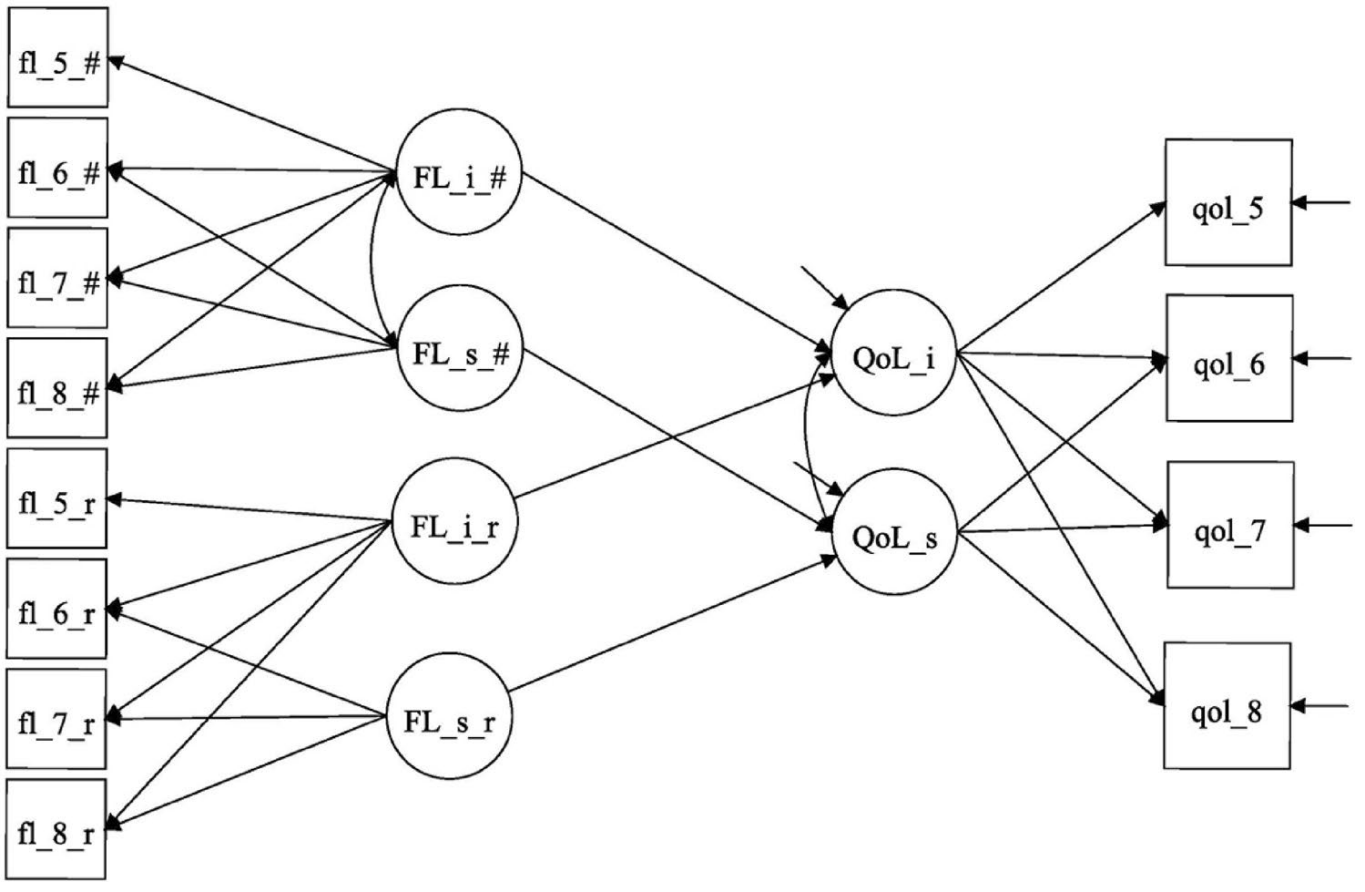
Outline of the latent growth model. QoL_i: Intercept for the latent variable Quality of Life QoL_s: Slope for the latent variable Quality of Life. qol_5 – qol_8: Observed indicators for the latent variable Quality of Life. FL_i_r: Intercept for the latent variable Risk of Functional Limitation. FL_s_r: Slope for the latent variable Risk of Functional Limitation. fl_5_r—fl_8_r: Observed indicators for the latent variable Risk of Functional Limitation. FL_i_#: Intercept for the latent variable Number of Functional Limitations. FL_s_#: Slope for the latent variable Number of Functional Limitations. fl_5_#—fl_8_#: Observed indicators for the latent variable Number of Functional Limitations.

First, the univariate descriptive statistics (i.e., means, standard deviations [SD], and percentages [%]) were produced to describe characteristics of middle-aged and older adults at baseline and at the three follow-ups, separately by multimorbidity status (with/without multimorbidity). To compare respondents in each age cohort by their morbidity status, we conducted a series of t-tests and chi-square tests for continuous and categorical variables, respectively. To estimate growth trajectories in quality of life and functional limitation in each age cohort and by multimorbidity status (i.e., separately in each of the four groups), we used latent growth curve (LGC) modeling techniques [38–40]. For the repeated continuous measures of quality of life, we specified a linear random (between persons) intercept and a random slope growth trajectory. Since over 70% of respondents reported no functional limitations across the seven-year study period, some of these respondents were assumed not to be at risk of experiencing any functional limitations (i.e., structural zeros). The repeated measures of functional limitation were treated as zero-inflated count variables and estimated as a zero-inflated Poisson (ZIP) growth trajectory with two components: an inflation component for respondents who were unable to assume any other counts except zero (i.e., not at risk of having any functional limitations during the study period) and a count component for respondents who were able to have counts of zero and above [41–43]. The latent growth curve models assume that the growth trajectories for all respondents in each of the four groups have the same functional form (i.e., linear for quality of life and Poisson for functional limitation), although individual growth trajectories are allowed to increase or decrease over time following these functional forms. These models also assume that the baseline covariates that affect the growth parameters affect respondents in each of the four groups in the same way. Figure 2 shows the outline of our generic LGC model.

To explore how multimorbidity moderates the relationship between growth trajectories in quality of life and functional limitation, we tested associations between growth parameters representing these two trajectories in a single model, separately in the two age cohorts and controlling for the baseline covariates. Taking into account smaller sample sizes for some groups and potential violation of the normality assumption, in all analyses, we used restricted maximum likelihood (MLR) with robust standard errors (sandwich estimator) and a numerical integration algorithm [44] to produce unbiased estimates of variance and covariance parameters. The calibrated individual sampling weights from the baseline survey allowed us to extrapolate the results to non-institutionalized residents of Luxembourg who were, in 2013, 50 years old and older. All data cleaning steps, including tests for model assumptions (i.e., normality, missing data, linearity, outliers), were conducted in SAS 9.4 [45] and all analyses were performed with Mplus 8 [46].

## Results

At baseline in 2013, 60.1% of all respondents had multimorbidity, 51.5% in the “middle age” cohort (with an average age of 57.9 years, 54.7% women) and 71.4% in the “older age” cohort (with an average age of 74.3 years, 50.4% women). The unweighted descriptive statistics for the repeated measures for quality of life, functional limitation, as well as for the baseline covariates are presented in Table 1.

**Table 1:** Descriptive statistics for quality of life, functional limitation, and control variables at baseline and the three follow-ups for middle-aged and older adults by multimorbidity status

	Time	All respondents			Middle age cohort								Older age cohort							
					With multimorbidity			Without multimorbidity			Difference		With multimorbidity			Without multimorbidity			Difference	
		*n*	Mean	SD	*n*	Mean	SD	*n*	Mean	SD	*t*-test	*p*-value	*n*	Mean	SD	*n*	Mean	SD	*t*-test	*p*-value
Quality of life	Baseline	1 500	39.7	5.5	441	38.1	6.0	422	41.1	4.5	8.25*	< .0001	455	39.1	5.5	182	41.8	4.3	6.57*	< .0001
	1st follow-up	1 046	39.7	5.5	303	38.5	5.6	298	41.1	4.6	6.19*	< .0001	329	38.8	5.9	116	41.6	4.5	5.32*	< .0001
	2nd follow-up	806	40.0	5.6	242	38.7	6.2	239	41.5	4.5	5.74*	< .0001	233	39.1	5.8	92	42.2	4.5	5.20*	< .0001
	3rd follow-up	596	40.2	5.4	180	38.8	6.2	187	41.8	4.0	5.45*	< .0001	163	39.2	5.6	66	42.4	4.0	4.82*	< .0001
No	baseline	463	2.1	2.9	127	1.6	1.9	49	1.1	1.5	1.85	0.0665	234	2.7	3.4	53	2.1	3.0	1.20	0.2733
Functional	1st follow-up	329	1.8	2.9	90	1.4	2.6	39	1.5	2.7	0.26	0.7966	167	2.2	3.2	33	1.7	2.8	0.99	0.3264
Limitations	2nd follow-up	265	1.8	2.8	82	1.5	2.5	34	1.4	2.1	0.11	0.9094	125	2.2	3.1	24	1.2	2.5	1.74	0.0896
	3rd follow-up	188	2.0	3.3	56	1.5	3.0	25	1.1	2.6	0.58	0.5664	87	2.7	3.7	20	1.7	2.6	1.34	0.1876

	Category	*n*	*%*		*n*	*%*		*n*	*%*		chi-square	*p*-value	*n*	*%*		*n*	*%*	chi-square	* p*-value	
Any limitations	No	1 279	80.7		375	81.2		406	93.3		29.44*	< 0.001	333	67.8		165	83.8	17.86*	< 0.0001	
(Baseline)	Yes	306	19.3		87	18.8		29	6.7				158	32.2		32	16.2			
Any limitations	No	912	83.8		275	86.8		289	94.1		9.79*	0.0018	241	70.3		107	87.7	14.54*	0.0001	
(1st follow-up)	Yes	177	16.3		42	13.3		18	5.9				102	29.7		15	12.3			
	Missing	496			145			128					148			75				
Any limitations	No	912	83.8		275	86.8		289	94.1		11.65*	0.0006	241	70.3		107	87.7	10.34*	0.0013	
(2nd follow-up)	Yes	146	17.4		45	17.9		19	7.7				71	28.5		11	11.8			
	Missing	745			211			188					242			104				
Any limitations	No	912	83.8		275	86.8		289	94.1		8.44*	0.0037	241	70.3		107	87.7	6.24*	0.0125	
(3rd follow-up)	Yes	99	15.3		25	13.0		9	4.6				55	28.8		10	13.9			
	Missing	936			270			241					300			125				
Gender	Males	747	47.1		210	45.5		196	45.1		0.01	0.9050	246	50.1		95	48.2	0.20	0.6560	
	Females	838	52.9		252	54.6		239	54.9				245	49.9		102	51.8			
Immigration	Born in Lux	1 036	65.4		274	59.3		263	60.5		0.12	0.7249	365	74.3		134	68.0	2.82	0.9330	
Status	Immigrant	549	34.6		188	40.7		172	39.5				126	25.7		63	32.0			
Having	No	374	23.6		93	20.1		62	14.3		5.41*	0.0200	158	32.2		61	31.0	0.10	0.7572	
Partner	Yes	1 211	76.4		369	79.9		373	85.8				333	67.8		136	69.0			
Educational	High	1 221	77.0		366	79.2		295	67.8		15.03*	0.0001	408	83.1		152	77.2	3.27	0.0704	
Attainment	Low	364	23.0		96	20.8		140	32.2				83	16.9		45	22.8			
Household	1st quartile	317	20.0		125	27.1		99	22.8		5.90	0.2070	69	14.1		24	12.2	2.18	0.7027	
Income	2nd quartile	317	20.0		91	19.7		75	17.2				108	22.0		43	21.8			
	3rd quartile	318	20.1		87	18.8		107	24.6				91	18.5		33	16.8			
	4th quartile	317	20.0		82	17.8		79	18.2				113	23.0		43	21.8			
	5th quartile	316	19.9		77	16.7		75	17.2				110	22.4		54	27.4			

Source: 2013–2020 SHARE data from Luxembourg (*n* = 1585)

*Statistically significant change across waves (*p* < 0.05)

*n*: Sample size

SD: Standard deviation

%: Percentage of respondents in each category

### Trajectories in quality of life and functional limitation

**Fig. 3:**
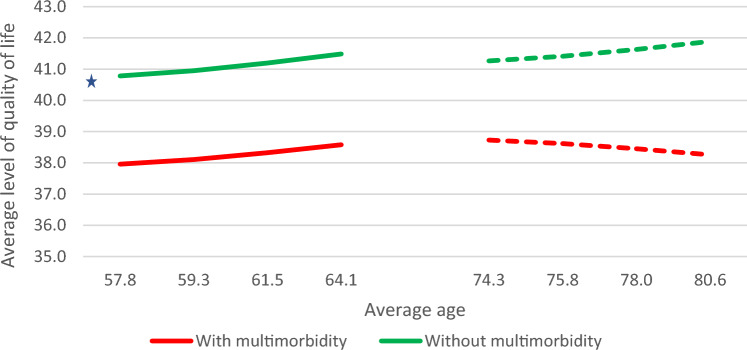
Model-estimated growth trajectory for quality of life across the life course by multimorbidity status (synthetic cohort). Source: 2013–2020 SHARE data from Luxembourg (*n* = 1,585). Solid line (—): Estimates from the cohort of middle-aged adults 50.0–64.9 years old at baseline (*n* = 897). Dotted line (---): Estimates from the cohort of older adults 65.0 years and over at baseline (*n* = 688). * Statistically significant change across waves (*p* < 0.05)

**Fig. 4:**
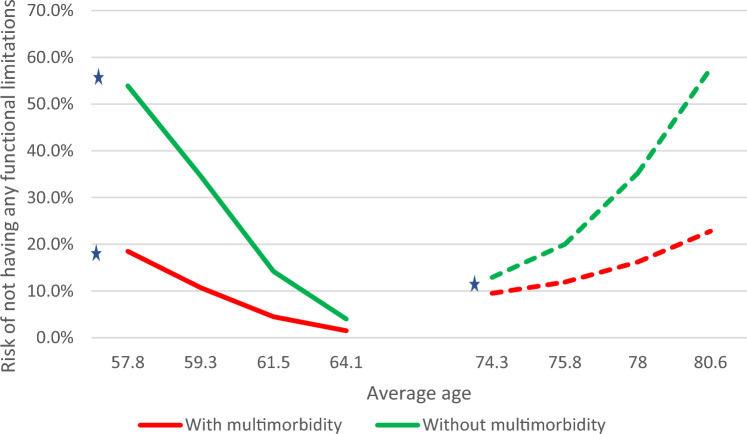
Model-estimated growth trajectory for the risk of not having any functional limitations across the life course by multimorbidity status (synthetic cohort). Source: 2013–2020 SHARE data from Luxembourg (*n* = 1,585). Solid line (—): Estimates from the cohort of middle-aged adults 50.0–64.9 years old at baseline (*n* = 897). Dotted line (---): Estimates from the cohort of older adults 65.0 years and older at baseline (*n* = 688). * Statistically significant change across waves (*p* < 0.05)

**Fig. 5:**
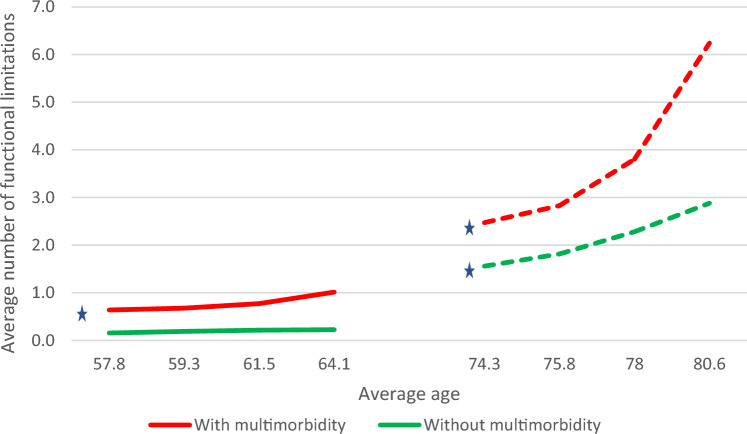
Model-estimated growth trajectory for the average number of functional limitations across the life course by multimorbidity status (synthetic cohort). Source: 2013–2020 SHARE data from Luxembourg (*n* = 1,585). Solid line (—): Estimates from the cohort of middle-aged adults 50.0–64.9 years old at baseline (*n* = 897). Dotted line (---): Estimates from the cohort of older adults 65.0 years and older at baseline (*n* = 688). * Statistically significant change across waves (*p* < 0.05)

Supplementary Table S2 presents the parameter estimates for growth trajectories in quality of life and functional limitation (i.e., means and variances of growth parameters, with their standard errors [SEs], p-values, and 95% confidence intervals [CIs]) for the two age cohorts and by multimorbidity status. These parameter estimates were converted into model estimated means and proportions for quality of life and functional limitation at baseline and at each follow-up (see Table 2). We also displayed these estimates in graphs, treating the two age cohorts as a synthetic cohort (see Figs. 3, 4, and 5) in order to allow for a more intuitive interpretation of the potential differences in impact of multimorbidity on quality of life and functional limitation across a longer span of the life course.

**Table 2:** Model estimated means and proportions for growth trajectories in quality of life and functional limitation at baseline and the three follow-ups for middle-aged and older adults by multimorbidity status

Age cohort and	Baseline survey	1st follow-up	2nd follow-up	3rd follow-up
Multimorbidity status	Wave 5 (2013)	Wave 6 (2015)	Wave 7 (2017)	Wave 8 (2019/20)
*Quality of life*				
Middle age cohort with multimorbidity	38.0	38.1	38.3	38.6
Middle age cohort without multimorbidity*	40.8	40.9	41.2	41.5
Cohen's D	0.54	0.56	0.54	0.57
Older age cohort with multimorbidity	38.7	38.6	38.5	38.3
Older age cohort without multimorbidity	41.3	41.4	41.6	41.9
Cohen's D	0.65	0.71	0.74	0.83
*Not at risk of functional limitation*				
Middle age cohort with multimorbidity*	18.5%	10.7%	4.5%	1.5%
Middle age cohort without multimorbidity	53.9%	34.6%	14.2%	4.0%
Older age cohort with multimorbidity*	9.5%	11.9%	16.2%	22.8%
Older age cohort without multimorbidity*	12.9%	20.0%	35.2%	57.6%
*Number of functional limitations*				
Middle age cohort with multimorbidity*	0.64	0.68	0.77	1.01
Middle age cohort without multimorbidity	0.16	0.19	0.22	0.22
Older age cohort with multimorbidity*	2.47	2.83	3.80	6.24
Older age cohort without multimorbidity*	1.56	1.81	2.28	2.88

**Source:** 2013–2020 SHARE data from Luxembourg (*n* = 1585)

*Statistically significant change across waves (*p* < 0.05)

At baseline, individuals living with multimorbidity had substantially lower levels of quality of life than individuals without multimorbidity (38.0 vs. 40.8 in the “middle age” cohort [Cohen’s D = 0.54] and 38.7 vs. 41.3 in the “older age” cohort [Cohen’s D = 0.65]; see Table 2). Assessing the change in the level of quality of life across the seven-year study period, only individuals without multimorbidity from the “middle age” cohort had a statistically significant increase in quality of life at a rate of 0.11 units per year (95% CI = 0.02–0.20). These cohort-level results suggest that the negative association between multimorbidity and quality of life observed at baseline remained relatively stable during the study period. Treating the group means as a synthetic cohort (see Fig. 3) suggests that there was a substantial negative impact of multimorbidity on quality of life that persisted throughout the life course from the age of 50.

We estimated proportions of middle-aged and older adults who were presumed not to be at risk of experiencing any functional limitations across the study period (structural zeros from the ZIP growth model) and means for the number of functional limitations for individuals at risk of experiencing any functional limitations (count component of the ZIP growth model). These estimates are presented in Table 2 and are displayed in the synthetic cohort format in Figs. 4 and 5, respectively. At baseline, when compared to adults with multimorbidity, a larger proportion of adults without multimorbidity was estimated not to be at risk of functional limitation (53.9% vs. 18.5% in the “middle age” cohort and 12.9% vs. 9.5% in the “older age” cohort). However, the impact of multimorbidity on this risk was more pronounced in the “middle age” cohort. The proportion of adults not at risk of functional limitation decreased over the study period in the “middle age” cohort with multimorbidity (from 18.5% to 1.5%) and increased in the “older age” cohort with multimorbidity (from 9.5% to 22.8%) and without multimorbidity (from 12.9% to 57.6%). When displayed as a synthetic cohort (see Fig. 4), these estimates suggest that the risk of having a functional limitation followed a non-linear trajectory, with a higher proportion of adults not being at risk at the start and end points of this trajectory.

In terms of the count component of the ZIP growth model, at baseline, middle-aged and older adults with multimorbidity had a higher number of functional limitations than adults without multimorbidity (0.64 vs. 0.16 in the “middle age” cohort and 2.47 vs. 1.56 in the “older age” cohort). In addition, our results imply that older adults had overall a higher number of functional limitations than middle-aged adults. The rate of increase in the average number of functional limitations over the study period was statistically significant in three groups: among middle-aged adults with multimorbidity (from 0.6 to 1.0) and among older adults with multimorbidity (from 2.5 to 6.2) and without multimorbidity (from 1.6 to 2.9). These estimates, when treated as a synthetic cohort (see Fig. 5), imply that individuals with multimorbidity had, on average, a larger number of functional limitations throughout the life course. However, this difference appeared to become more pronounced as adults progressed from the middle-aged to older stage of the life course.

### Longitudinal relationship between functional limitation and quality of life

The results for the impact of multimorbidity on the relationship between trajectories in quality of life and functional limitation in each of the four groups are presented in Table 3. Supplementary Table S3 also presents the parameter estimates for the associations between control variables and the growth parameters. At baseline, a higher number of functional limitations was associated with poorer quality of life in all groups. This association, however, was stronger among individuals living with multimorbidity. Among middle-aged adults, an additional functional limitation was associated with a decline in quality of life by 1.02 units (95% CI: -1.34 to -0.70) for individuals with multimorbidity, compared to a less pronounced decline of 0.54 units (95% CI: − 0.86 to − 0.21) for individuals without multimorbidity. Among older adults, one additional functional limitation was associated with a decrease in quality of life by 1.80 units (95% CI: − 2.20 to − 1.40) with multimorbidity and by 1.23 units (95% CI: − 1.77 to − 0.69) for individuals without multimorbidity.

**Table 3:** Relationships between parameter estimates for growth trajectories for quality of life and number of functional limitations for middle-aged and older adults by multimorbidity status

	With multimorbidity					Without multimorbidity				
	Estimate	SE	*p*-value	Lower CI	Upper CI	Estimate	SE	*p*-value	Lower CI	Upper CI
*Middle age cohort (age 50.0–64.9 at baseline; n* = *897)*										
QoL_i <—FL_i_#	− 1.02	0.16	0.00	− 1.34	− 0.70	− 0.54	0.17	0.00	− 0.86	− 0.21
QoL_s <—FL_s_#	− 3.27	3.55	0.36	− 10.23	3.68	− 1.59	0.66	0.02	− 2.89	− 0.29
QoL_s <—> QoL_i	− 0.47	0.41	0.26	− 1.26	0.34	− 0.91	0.30	0.00	− 1.50	− 0.31
*Means*										
QoL_i	34.81	0.83	0.00	33.19	36.43	37.70	0.96	0.00	35.82	39.58
QoL_s	0.17	0.23	0.46	− 0.29	0.63	− 0.19	0.28	0.51	− 0.73	0.36
FL_i_#	− 2.40	0.48	0.00	− 3.34	− 1.46	− 3.31	1.03	0.00	− 5.32	− 1.29
FL_s_#	0.00	0.07	0.97	− 0.14	0.14	− 0.24	0.14	0.09	− 0.51	0.04
*Variances*										
QoL_i	17.13	2.16	0.00	12.90	21.35	13.91	1.72	0.00	10.54	17.27
QoL_s	0.18	0.28	0.52	− 0.37	0.73	0.08	0.10	0.43	− 0.12	0.28
FL_i_#	4.29	0.62	0.00	3.07	5.51	4.38	1.08	0.00	2.27	6.50
FL_s_#	0.02	0.02	0.36	− 0.02	0.07	0.05	0.04	0.23	− 0.03	0.14
*Older age cohort (age 65.0 and older at baseline; n* = *688)*										
QoL_i <—FL_i_#	− 1.80	0.20	0.00	− 2.20	− 1.40	− 1.23	0.28	0.00	− 1.77	− 0.69
QoL_s <—FL_s_#	− 2.02	0.80	0.01	− 3.59	− 0.46	− 1.65	1.10	0.13	− 3.82	0.51
QoL_s <—> QoL_i	− 0.29	0.42	0.49	− 1.10	0.53	− 0.05	0.35	0.90	− 0.74	0.65
*Means*										
QoL_i	36.40	0.66	0.00	35.10	37.69	36.73	1.11	0.00	34.56	38.90
QoL_s	− 0.10	0.19	0.59	− 0.47	0.27	0.55	0.52	0.29	− 0.46	1.56
FL_i_#	− 0.73	0.29	0.01	− 1.29	− 0.17	− 2.85	0.62	0.00	− 4.06	− 1.64
FL_s_#	0.12	0.05	0.03	0.01	0.22	0.28	0.19	0.14	− 0.09	0.66
*Variances*										
QoL_i	13.96	2.27	0.00	9.51	18.41	6.35	1.90	0.00	2.63	10.07
QoL_s	0.13	0.15	0.38	− 0.16	0.42	− 0.11	0.19	0.57	− 0.47	0.26
FL_i_#	2.40	0.32	0.00	1.78	3.03	3.10	0.99	0.00	1.16	5.05
FL_s_#	0.03	0.01	0.01	0.01	0.06	0.08	0.05	0.14	− 0.03	0.18

**Source**: 2013–2020 SHARE data from Luxembourg (*n* = 1585)

QoL_i: Intercept for the latent variable Quality of Life

QoL_s: Slope for the latent variable Quality of Life

FL_i_#: Intercept for the latent variable Number of Functional Limitations

FL_s_#: Slope for the latent variable Number of Functional Limitations

SE: Standard error

CI: 95% confidence interval

Regarding the relationship between change in the number of functional limitations and change in the level of quality of life, the direction and magnitude of parameter estimates for the four groups suggested that an increase in the number of functional limitations over the seven-year study period was associated with a decline in quality of life. This association was stronger among individuals with multimorbidity. However, the parameter estimates for these effects were only statistically significant in two groups: among middle-aged adults without multimorbidity, one additional functional limitation was associated with a decrease in quality of life by 1.59 units (95% CI: − 2.89 to − 0.29) and by 2.02 units (95% CI: − 3.59 to − 0.46) among older adults with multimorbidity. The parameter estimates for the other two groups, i.e., middle-aged adults with multimorbidity (b = − 3.27, 95% CI: − 10.23 to 3.68) and older adults without multimorbidity (b = − 1.65, 95% CI: − 3.82 to 0.51), were not statistically significant. This was likely due to substantial variation in these effects within each group.

## Discussion

As an increasing number of individuals in Europe and in other parts of the world are becoming vulnerable to more years lived with multiple chronic health conditions, there is a growing need to identify factors that might lead to improvements in or maintenance of quality of life among individuals living with multimorbidity. This study assessed how multimorbidity affects trajectories in functional limitation and quality of life as well as the relationship between these trajectories. We also explored whether these relationships vary across the life course.

Compared to previous cross-sectional studies [5, 8–11], we took advantage of the longitudinal SHARE data and a multi-cohort study design. We found that middle-aged and older adults living with multimorbidity exhibited consistently poorer quality of life than those living without multimorbidity throughout the life course. In addition, while supporting past findings that individuals living with multimorbidity have a higher degree of functional limitation [11, 19–22], our study contributed to a more refined understanding of the nature of this relationship. Specifically, we found that, throughout the life course, a larger proportion of adults living with multimorbidity was at a higher risk of experiencing a functional limitation than those without multimorbidity. We also revealed that middle-aged and older adults living with multimorbidity had a higher number of functional limitations compared to those without multimorbidity. This gap may be larger among older adults.

Our study emphasized the need to assess how multimorbidity might affect the longitudinal relationship between the number of functional limitations and quality of life. At baseline, functional limitation had an overall negative impact on quality of life, as previously suggested in a number of cross-sectional studies [11, 13, 15, 47], and this effect was stronger among individuals with multimorbidity. This confirmed our hypothesis that multimorbidity is likely to exacerbate the relationship between functional limitation and quality of life. The longitudinal design of our study allowed us to provide some support for the hypothesis that an increase in the number of functional limitations leads to a decline in quality of life over time. However, this association was statistically significant among middle-aged adults without multimorbidity and among older adults with multimorbidity. The direction and magnitude of the significant and non-significant parameter estimates suggested that the negative impact of an increase in functional limitation on the decline in quality of life is more pronounced among individuals living with multimorbidity. This observation, however, would have to be tested and confirmed in additional longitudinal studies.

This study was based on earlier conceptualizations of the interrelationships between multimorbidity, functional limitation, and quality of life. Using a cross-sectional sample of adults 65 years and older from the Medicare Health Outcomes Survey, Barile and colleagues found that the association between the number of functional limitations and quality of life was moderated by the number of chronic conditions, i.e., multimorbidity [15]. In a cross-sectional study of SHARE respondents from several European settings, Makovski and colleagues highlighted that the number of chronic conditions and functional limitations had an independent relationship with quality of life [13]. To the best of our knowledge, our study is the first longitudinal assessment of these relationships. It allowed us to conclude that the previously observed cross-sectional associations of multimorbidity with functional limitation and quality of life are more persistent and can affect middle-aged and older adults over the life course. However, this relationship was not statistically significant in all groups and needs to be further investigated. Finally, our results suggest that the strength of the association of multimorbidity with functional limitation and quality of life, as well as the relationship between these two health outcomes, may differ across the life course. This initial observation, however, would have to be formally tested and confirmed in additional longitudinal studies. This novel finding suggests that any future research on the interrelationship between multimorbidity, functional limitation, and quality of life should assess these relationships separately for middle-aged and older adults.

This study also employed a number of novel methodological techniques that may be beneficial to future research on healthy ageing. Firstly, the longitudinal study design and LGC modeling techniques offered a unique opportunity not only to assess the trajectories in individual health outcomes but also the relationships between these trajectories. Secondly, taking advantage of the multi-cohort study design, we explored how multimorbidity might affect functional limitation and quality of life across a longer span of the life course than a single age cohort would allow us to do. However, we suggest that future studies with longer follow-up time periods would be in a better position to untangle complex interrelationships between multimorbidity, functional limitation, and quality of life compared to studies relying only on synthetic cohorts. Thirdly, in the past, the ADL/IADL summated scale was used either as a binary indicator [13, 21, 23], count variable [19], or as a continuous scale [15]. We proposed a more realistic measurement option for functional limitation by assuming the existence of two longitudinal processes, the over-time change in the risk of having functional limitation and, among those at risk, the change in the number of limitations. We encourage future studies to provide further validation for this modeling approach. Finally, a number of past studies indicated that there exist substantial across-country differences in the prevalence of and relationships between multimorbidity, functional limitation, and quality of life. This suggests that demographic, socioeconomic, and health care system differences across contexts have pronounced influences on these relationships [5, 13, 22]. Thus, although there are some advantages in using pooled data from multiple geographic settings, we employed a more homogeneous sample of non-institutionalized residents of Luxembourg. We encourage future research to corroborate our findings in other contexts.

Some limitations of our study warrant mentioning. A selection bias could have affected the representativeness of the sample, as individuals with poorer health or lower socioeconomic status tend to be under-represented in self-reported population surveys and are more likely to drop out throughout the study period. To address this issue, we used sampling weights, controlled for key indicators of socioeconomic status, and introduced statistical adjustments for attrition and missing data. Our measures of chronic conditions, functional limitation, and quality of life were self-reported and could be affected by recall bias. However, self-reports are commonly used in large population-based studies [48] and a sensitivity analysis of SHARE data has indicated a very strong concordance in reporting the same chronic conditions across time [22]. Future research should explore how specific sub-domains of the multidimensional CASP-12 scale (i.e., control, autonomy, self-realization, and pleasure) as well as other aspects of quality of life (e.g., social and functional aspects, mental health, vitality) are associated with multimorbidity and functional limitation.

Our study focused on the role of multimorbidity. Past research, however, has provided some evidence that distinct patterns and combinations of chronic conditions may be differentially associated with functional limitation [9, 23, 49]. Thus, future studies should consider assessing the relative role of specific chronic conditions or clusters of conditions (e.g., musculoskeletal, cardiometabolic, and mental health) in functional limitation and quality of life among people living with multimorbidity. Future studies should also assess how time of onset and progression of chronic conditions across the life course may lead to differential trajectories in quality of life and functional limitation.

An important limitation of the study is the lack of information on additional confounding variables. These would include major modifiable risk factors (e.g., smoking, alcohol consumption, diet, physical activity, sleep), which are known to influence both multimorbidity risk and the selected study outcomes. SHARE data focuses on individuals ≥ 50 years and excludes those between the ages of 45 and 49, limiting our ability to assess the relationship between multimorbidity, functional limitation, and quality of life across the whole spectrum of “middle age”, as defined by Erikson [30].

Furthermore, length of the follow-up in our study could be too short to detect long-term effects of multimorbidity on functional limitation and quality of life. Although we took advantage of the multi-cohort design and presented our results as a synthetic cohort, these results should be treated as exploratory and confirmed in studies with longer follow-ups. Finally, the statistically significant variances associated with the parameter estimates for the growth trajectories suggested that there is a substantial degree of heterogeneity across person-specific functional limitation and quality of life trajectories. Although our study did not explore the presence of latent classes consisting of individuals with more homogenous growth trajectories, this might be a potentially fruitful direction for future research on healthy ageing. It may also be beneficial to identify sub-groups of individuals living with multimorbidity, as these groups may have different needs and may require more targeted interventions to address their functional limitations and improve their quality of life.

## Conclusion

Improving or maintaining a good quality of life can be an important coping mechanism for individuals living with multimorbidity [50]. The results from the current study contribute to a better understanding of the complexity of the relationship between multimorbidity, functional limitation, and quality of life by focusing on how these three health outcomes interact across the life course. However, additional longitudinal research from multiple geographic contexts and with longer follow-up time periods is needed to further explore the underlying mechanisms of these interrelationships. This includes an assessment of the mediating role of functional limitation in the relationship between multimorbidity and quality of life as well as on identification of other factors that may mediate or moderate these relationships. From a clinical standpoint, health professionals should pay great attention to the clinical management of people living with multimorbidity via a patient-centred approach, in order to mitigate the potential negative impact of co-occurring multiple chronic conditions on functional limitation and quality of life over time [51].

### Supplementary Information

Below is the link to the electronic supplementary material.

Supplementary file1 (PDF 4112 KB)

## Data Availability

Share data is available to all the scientific community upon request at https://share-eric.eu/data/.
