# Supplementary material for: Relationship between multimorbidity, functional limitation, and quality of life among middle-aged and older adults: findings from the longitudinal analysis of the 2013–2020 Survey of Health, Ageing, and Retirement in Europe (SHARE)
Source: Qual Life Res. 2023 Sep 30;33(1):169–81. doi: 10.1007/s11136-023-03508-9 (PMC10784342; doi:10.1007/s11136-023-03508-9)
Supplement: Supplementary file 1 — Supplementary file1 (PDF 4112 KB) [file 11136_2023_3508_MOESM1_ESM.pdf]

Supplementary Tables

Table S1: Descriptive statistics for quality of life, functional limitation, and control variables at baseline by survey response pattern.

		All respondents			Participated in 3 follow-ups			Did not participate in all follow-ups			Difference
	Time	*n*	*Mean*	*Std Dev*	*n*	*Mean*	*Std Dev*	*n*	*Mean*	*Std Dev*	*t-test*
Quality of life	baseline	1,500	39.7	5.5	527	40.0	5.3	973	39.5	5.6	1.93
Number of functional limitations	baseline	463	2.1	2.9	159	1.2	2.0	304	2.6	3.2	4.98 *
	Category	*n*	*%*		*n*	*%*		*n*	*%*		*chi-square*
Any limitations	no	1,279	80.7		467	86.0		812	77.9		14.95 *
	yes	306	19.3		76	14.0		230	22.1		
Gender	males	747	47.1		244	44.9		503	48.3		1.60
	females	838	52.9		299	55.1		539	51.7		
Immigration status	born in Lux.	1,036	65.4		369	68.0		667	64.0		2.45
	immigrant	549	34.6		174	32.0		375	36.0		
Having partner	no	374	23.6		111	20.4		263	25.2		4.56 *
	yes	1,211	76.4		432	79.6		779	74.8		
Educational attainment	high	1,221	77.0		400	73.7		821	78.8		5.30 *
	low	364	23.0		143	26.3		221	21.2		
Household income	1st quartile	317	20.0		99	18.2		218	20.9		3.15
	2nd quartile	317	20.0		107	19.7		210	20.1		
	3rd quartile	318	20.1		105	19.3		213	20.4		
	4th quartile	317	20.0		116	21.4		201	19.3		
	5th quartile	316	19.9		116	21.4		200	19.2		

Source: 2013-2020 SHARE data from Luxembourg (n=1,585)

* Statistically significant change across waves ($p < 0.05$)

Table S2: Parameter estimates for growth trajectories in quality of life and functional limitation limitations for middle-aged and older adults by multimorbidity status.

	With multimorbidity					Without multimorbidity				
**Quality of life**	**Estimate**	**SE**	**p-value**	**Lower CI**	**Upper CI**	**Estimate**	**SE**	**p-value**	**Lower CI**	**Upper CI**
Middle age cohort (age 50.0-64.9 at baseline; n=897)										
Means:										
QoL_i	37.96	0.29	0.00	37.39	38.53	40.78	0.25	0.00	40.30	41.26
QoL_s	0.10	0.07	0.13	-0.03	0.23	0.11	0.05	0.02	0.02	0.20
Variances:										
QoL_i	24.60	2.56	0.00	19.58	29.61	16.24	1.94	0.00	12.44	20.05
QoL_s	0.41	0.13	0.00	0.16	0.66	0.23	0.07	0.00	0.09	0.38
QoL_s <-> QoL_i	-0.66	0.46	0.15	-1.55	0.23	-1.09	0.31	0.00	-1.70	-0.47
Older age cohort (Age 65 and older at baseline)										
Means:										
QoL_i	38.73	0.28	0.00	38.19	39.27	41.26	0.35	0.00	40.58	41.94
QoL_s	-0.08	0.07	0.28	-0.21	0.06	0.10	0.08	0.19	-0.05	0.25
Variances:										
QoL_i	22.39	2.68	0.00	17.14	27.65	12.36	2.20	0.00	8.04	16.67
QoL_s	0.36	0.14	0.01	0.09	0.63	0.03	0.13	0.80	-0.22	0.29
QoL_s <-> QoL_i	-0.48	0.49	0.33	-1.43	0.47	-0.14	0.42	0.74	-0.97	0.68
**Functional limitation**	**Estimate**	**SE**	**p-value**	**Lower CI**	**Upper CI**	**Estimate**	**SE**	**p-value**	**Lower CI**	**Upper CI**
Middle age cohort (Age 50-64 at baseline)										
Means:										
FL_i_r	—	—	—	—	—	—	—	—	—	—
FL_s_r	-0.42	0.12	0.00	-0.67	-0.18	-0.53	0.36	0.14	-1.23	0.17
FL_i_#	-2.53	0.25	0.00	-3.02	-2.03	-3.33	0.38	0.00	-4.07	-2.58
FL_s_#	-0.15	0.08	0.05	-0.30	0.00	-0.39	0.14	0.01	-0.66	-0.12
Variances:										
FL_i_r	—	—	—	—	—	—	—	—	—	—
FL_s_r	—	—	—	—	—	—	—	—	—	—
FL_i_#	4.57	0.59	0.00	3.41	5.73	4.48	0.70	0.00	3.11	5.85
FL_s_#	0.03	0.01	0.00	0.01	0.05	0.02	0.02	0.29	-0.01	0.05
covariance (count)	0.11	0.08	0.19	-0.05	0.27	0.28	0.12	0.02	0.05	0.51
Older age cohort (age 65.0 and older at baseline; n=688)										
Means:										
FL_i_r	—	—	—	—	—	—	—	—	—	—
FL_s_r	0.16	0.07	0.03	0.02	0.31	0.35	0.11	0.00	0.13	0.58
FL_i_#	-1.14	0.09	0.00	-1.31	-0.97	-2.82	0.33	0.00	-3.46	-2.18
FL_s_#	0.14	0.03	0.00	0.09	0.19	0.24	0.12	0.05	0.00	0.48
Variances:										
FL_i_r	—	—	—	—	—	—	—	—	—	—
FL_s_r	—	—	—	—	—	—	—	—	—	—
FL_i_#	4.29	0.23	0.00	3.85	4.73	6.82	0.96	0.00	4.93	8.71
FL_s_#	0.03	0.00	0.00	0.02	0.03	0.02	0.01	0.00	0.01	0.04
FL_s_# <-> FL_i_#	-0.05	0.04	0.23	-0.14	0.03	-0.10	0.26	0.70	-0.61	0.41

Source: 2013-2020 SHARE data from Luxembourg (n=1,585)

QoL_i: Intercept for the latent variable Quality of Life

QoL_s: Slope for the latent variable Quality of Life

FL_i_r: Intercept for the latent variable Risk of Functional Limitation

FL_s_r: Slope for the latent variable Risk of Functional Limitation

FL_i_#: Intercept for the latent variable Number of Functional Limitations

FL_s_#: Slope for the latent variable Number of Functional Limitations

SE: Standard error

CI: 95% confidence interval

Table S3: Parameter estimates for the associations between control variables and the growth parameters for middle-aged and older adults by multimorbidity status.

	With multimorbidity					Without multimorbidity				
Middle age cohort (age 50.0-64.9 at baseline; n=897)										
	**Estimate**	**SE**	**p-value**	**Lower CI**	**Upper CI**	**Estimate**	**SE**	**p-value**	**Lower CI**	**Upper CI**
QoL_i <- FL_i_#	-1.02	0.16	0.00	-1.34	-0.70	-0.54	0.17	0.00	-0.86	-0.21
QoL_s <- FL_s_#	-3.27	3.55	0.36	-10.23	3.68	-1.59	0.66	0.02	-2.89	-0.29
QoL_s <-> QoL_i	-0.47	0.41	0.26	-1.26	0.34	-0.91	0.30	0.00	-1.50	-0.31
Means:										
QoL_i	34.81	0.83	0.00	33.19	36.43	37.70	0.96	0.00	35.82	39.58
QoL_s	0.17	0.23	0.46	-0.29	0.63	-0.19	0.28	0.51	-0.73	0.36
FL_i_#	-2.40	0.48	0.00	-3.34	-1.46	-3.31	1.03	0.00	-5.32	-1.29
FL_s_#	0.00	0.07	0.97	-0.14	0.14	-0.24	0.14	0.09	-0.51	0.04
Variances:										
QoL_i	17.13	2.16	0.00	12.90	21.35	13.91	1.72	0.00	10.54	17.27
QoL_s	0.18	0.28	0.52	-0.37	0.73	0.08	0.10	0.43	-0.12	0.28
FL_i_r	4.29	0.62	0.00	3.07	5.51	4.38	1.08	0.00	2.27	6.50
FL_s_r	0.02	0.02	0.36	-0.02	0.07	0.05	0.04	0.23	-0.03	0.14
QoL_i <-										
Age	0.00	0.07	0.99	-0.14	0.14	0.03	0.06	0.64	-0.09	0.15
Gender	0.37	0.57	0.51	-0.74	1.48	1.06	0.53	0.05	0.02	2.11
Immigrant	-1.51	0.57	0.01	-2.63	-0.39	-1.53	0.53	0.00	-2.57	-0.50
Education	1.22	0.67	0.07	-0.09	2.54	1.15	0.55	0.04	0.08	2.22
Income	0.24	0.19	0.20	-0.13	0.60	0.15	0.18	0.40	-0.20	0.49
Partner	0.73	0.59	0.22	-0.43	1.88	1.19	0.67	0.07	-0.12	2.50
QoL_s <-										
Age	-0.03	0.03	0.46	-0.09	0.04	0.00	0.02	0.87	-0.04	0.05
Gender	-0.29	0.22	0.20	-0.73	0.15	-0.35	0.18	0.06	-0.70	0.01
Immigrant	-0.32	0.46	0.49	-1.22	0.59	0.29	0.22	0.20	-0.15	0.73
Education	-0.31	0.71	0.66	-1.70	1.08	-0.57	0.32	0.08	-1.19	0.06
FL_i_#										
Age	0.06	0.04	0.13	-0.02	0.15	0.00	0.06	0.98	-0.12	0.12
Gender	0.67	0.33	0.04	0.03	1.30	1.15	0.54	0.03	0.10	2.20
Immigrant	0.25	0.32	0.44	-0.38	0.88	-0.74	0.49	0.13	-1.70	0.22
Education	-1.05	0.48	0.03	-1.99	-0.11	-0.05	0.56	0.93	-1.14	1.04
Income	-0.03	0.12	0.77	-0.26	0.19	-0.08	0.16	0.64	-0.39	0.24
Partner	-0.75	0.35	0.03	-1.44	-0.06	-0.46	0.55	0.40	-1.55	0.62
FL_s_#										
Age	0.00	0.01	0.79	-0.02	0.02	0.01	0.02	0.34	-0.02	0.04
Gender	-0.01	0.07	0.86	-0.14	0.12	-0.15	0.14	0.27	-0.42	0.12
Immigrant	-0.14	0.07	0.07	-0.28	0.01	0.09	0.14	0.54	-0.19	0.35
Education	-0.06	0.16	0.70	-0.37	0.25	-0.19	0.17	0.27	-0.53	0.15
Older age cohort (age 65.0 and older at baseline; n=688)										
	**Estimate**	**SE**	**p-value**	**Lower CI**	**Upper CI**	**Estimate**	**SE**	**p-value**	**Lower CI**	**Upper CI**
QoL_i <- FL_i_#	-1.80	0.20	0.00	-2.20	-1.40	-1.23	0.28	0.00	-1.77	-0.69
QoL_s <- FL_s_#	-2.02	0.80	0.01	-3.59	-0.46	-1.65	1.10	0.13	-3.82	0.51
QoL_s <-> QoL_i	-0.29	0.42	0.49	-1.10	0.53	-0.05	0.35	0.90	-0.74	0.65
Means:										
QoL_i	36.40	0.66	0.00	35.10	37.69	36.73	1.11	0.00	34.56	38.90
QoL_s	-0.10	0.19	0.59	-0.47	0.27	0.55	0.52	0.29	-0.46	1.56
FL_i_#	-0.73	0.29	0.01	-1.29	-0.17	-2.85	0.62	0.00	-4.06	-1.64
FL_s_#	0.12	0.05	0.03	0.01	0.22	0.28	0.19	0.14	-0.09	0.66
Variances:										
QoL_i	13.96	2.27	0.00	9.51	18.41	6.35	1.90	0.00	2.63	10.07
QoL_s	0.13	0.15	0.38	-0.16	0.42	-0.11	0.19	0.57	-0.47	0.26
FL_i_#	2.40	0.32	0.00	1.78	3.03	3.10	0.99	0.00	1.16	5.05
FL_s_#	0.03	0.01	0.01	0.01	0.06	0.08	0.05	0.14	-0.03	0.18
QoL_i <-										
Age	0.17	0.04	0.00	0.09	0.26	0.13	0.07	0.05	0.00	0.26
Gender	0.65	0.54	0.23	-0.41	1.71	1.11	0.82	0.18	-0.50	2.72
Immigrant	-1.38	0.65	0.04	-2.65	-0.10	-0.35	0.88	0.69	-2.07	1.38
Education	0.44	0.71	0.54	-0.95	1.82	0.65	1.04	0.53	-1.39	2.69
Income	-0.19	0.18	0.30	-0.54	0.16	0.14	0.28	0.63	-0.42	0.69
Partner	0.81	0.53	0.12	-0.22	1.84	1.03	0.78	0.19	-0.49	2.55
QoL_s <-										
Age	-0.01	0.02	0.39	-0.04	0.02	0.02	0.02	0.32	-0.02	0.06
Gender	0.08	0.15	0.61	-0.21	0.36	-0.23	0.34	0.50	-0.89	0.43
Immigrant	0.00	0.17	0.99	-0.34	0.34	-0.23	0.38	0.54	-0.97	0.51
Education	0.00	0.21	0.99	-0.41	0.40	-0.72	0.48	0.14	-1.66	0.23
FL_i_#										
Age	0.11	0.01	0.00	0.08	0.13	0.18	0.03	0.00	0.12	0.24
Gender	0.20	0.23	0.39	-0.26	0.65	0.43	0.51	0.40	-0.56	1.42
Immigrant	-0.11	0.26	0.67	-0.63	0.40	0.13	0.65	0.84	-1.14	1.39
Education	-0.44	0.36	0.23	-1.15	0.28	-0.16	0.67	0.81	-1.49	1.16
Income	-0.22	0.07	0.00	-0.36	-0.08	-0.26	0.18	0.14	-0.60	0.09
Partner	-0.44	0.22	0.04	-0.86	-0.01	-0.11	0.49	0.82	-1.06	0.84
FL_s_#										
Age	0.01	0.00	0.00	0.01	0.02	0.01	0.01	0.42	-0.01	0.03
Gender	-0.06	0.05	0.23	-0.17	0.04	-0.17	0.16	0.27	-0.48	0.13
Immigrant	0.03	0.07	0.61	-0.10	0.16	-0.04	0.24	0.87	-0.51	0.43
Education	-0.06	0.09	0.53	-0.23	0.12	-0.39	0.22	0.08	-0.83	0.04

Source: 2013-2020 SHARE data from Luxembourg (n=1,585)

QoL_i: Intercept for the latent variable Quality of Life

QoL_s: Slope for the latent variable Quality of Life

FL_i_r: Intercept for the latent variable Risk of Functional Limitation

FL_s_r: Slope for the latent variable Risk of Functional Limitation

FL_i_#: Intercept for the latent variable Number of Functional Limitations

FL_s_#: Slope for the latent variable Number of Functional Limitations

SE: Standard error

CI: 95% confidence interval
